# Severe varicella in a child immunosuppressed with methotrexate^☆^

**DOI:** 10.1016/j.abd.2020.10.017

**Published:** 2022-02-08

**Authors:** Letícia Oba Galvão, Carmelia Matos Santiago Reis, Natália Lima Alves, Elisa Scandiuzzi Maciel

**Affiliations:** a Dermatology Service, Hospital Regional da Asa Norte, Brasília, DF, Brazil; b Escola Paulista de Medicina, Universidade Federal de São Paulo, São Paulo, SP, Brazil

**Keywords:** Immunosuppression, Methotrexate, Psoriasis, Varicella

## Abstract

Varicella is a common viral infection in childhood and usually has a benign evolution. However, the evolution can be severe in immunosuppressed children. Vaccination can prevent its occurrence and especially the development of severe and/or complicated cases. Methotrexate is a systemic therapeutic option for many inflammatory diseases, and its immunosuppressive action exposes its users to a higher susceptibility to infection. The present report describes a case of severe varicella infection in a child immunosuppressed with methotrexate.

## Introduction

Varicella, or chickenpox, is a common viral infection in childhood, characterized by a vesicular eruption. In immunocompetent individuals, the symptoms are usually mild to moderate, but uncomplicated severe cases may present with more than 1,000 lesions and severe constitutional symptoms.^1^ Severe complications, including central nervous system involvement, pneumonia, secondary bacterial infections, and death,^2^ have been described, especially in immunocompromised patients.^3^ Vaccination can prevent its occurrence and especially the development of severe and complicated conditions.^4^

Psoriasis is a chronic inflammatory disease that significantly compromises the quality of life, requiring effective treatment. Up to one-third of psoriasis cases start in childhood and, although most children have moderate disease, which is well controlled with topical therapy, up to 20% of these children require systemic therapy.^5^

Methotrexate is a cost-effective systemic therapy option for several inflammatory diseases, available in the Brazilian Unified Health System (SUS, *Sistema Único de Saúde*). However, its immunosuppressive action exposes its users to increased susceptibility to infections.^6^ The pre-methotrexate assessment includes blood count, glucose measurement, liver and kidney enzymes, serology for hepatitis B and C, human immunodeficiency virus (HIV), and chest X-ray.^7^ This screening includes the main contraindications of the medication, such as immunodeficiency, presence of active infection, hematological, renal, and hepatic alterations.^7^ However, updating of the vaccination card is not part of the routine for starting methotrexate therapy.

This case report aims to draw attention to the increased susceptibility and severity of opportunistic infections in immunosuppressed children and also to alert to the need to update the vaccination card in individuals submitted to immunosuppressive therapy.

## Case report

A 12-year-old female child had erythrodermic psoriasis for two years, controlled with methotrexate, 15 mg per week, which was started one year and five months before. With psoriasis under control, she was scheduled to withdraw the medication, but she returned to consultation reporting the onset of painful bulla and vesicles on the neck for five days, which after one day spread to the upper limbs, trunk, face, and oral mucosa, leading to difficulty in swallowing. The mother reported the child had had fever which was not measured.

She had a personal history of allergic rhinitis, denied episodes of varicella, reported contact with a child with active varicella lesions, and, when evaluating the child's vaccination card, no previous vaccination for this disease was identified.

**Figure 1:**
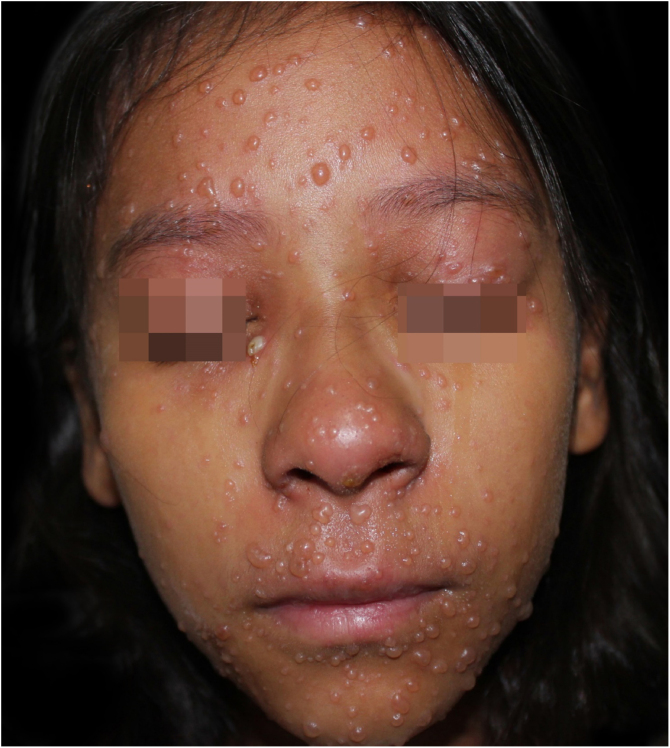
At hospital admission, on the fifth day of disease evolution, with vesicles and bullae on the face and a pustule on the right lower eyelid.

On physical examination, she showed regular general status, hypoactive, with a temperature of 39.8 °C, with numerous tense and confluent vesicles and bulla on the face, oral mucosa, neck, trunk, and upper limbs, which were more sparse in the lower limbs, where some lesions showed hematic content (Fig. 1). She had whitish plaques throughout her oral cavity.

With the hypothesis of varicella, she was promptly admitted to the hospital, and a biopsy was performed for histopathological confirmation. Home-administered medications were withdrawn, and therapy was started with intravenous acyclovir 10 mg/kg/day, and oral nystatin, in addition to bathing with potassium permanganate solution (1:4 liters). The anatomopathological analysis later confirmed the diagnosis of varicella.

**Figure 2:**
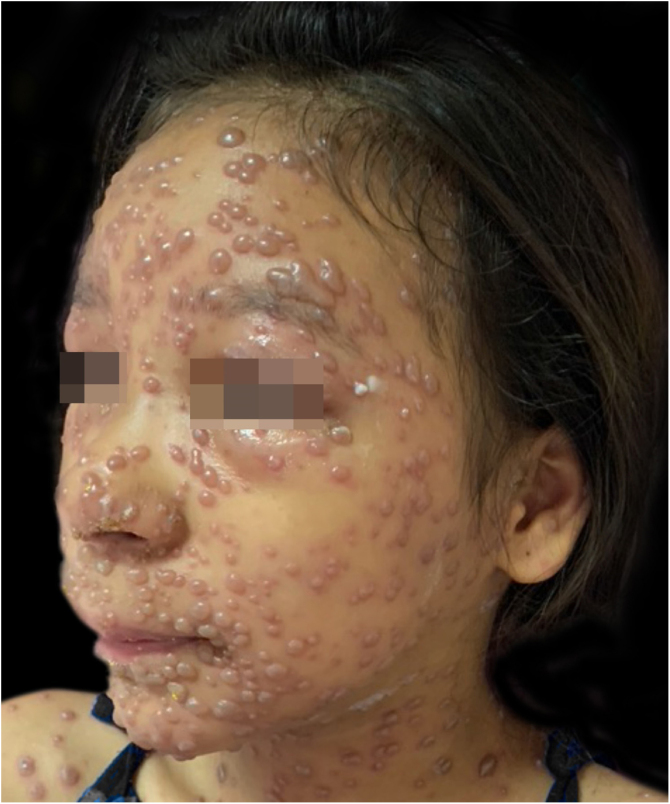
At the seventh day of evolution, presenting a bullous eruption with an erythematous background.

**Figure 3:**
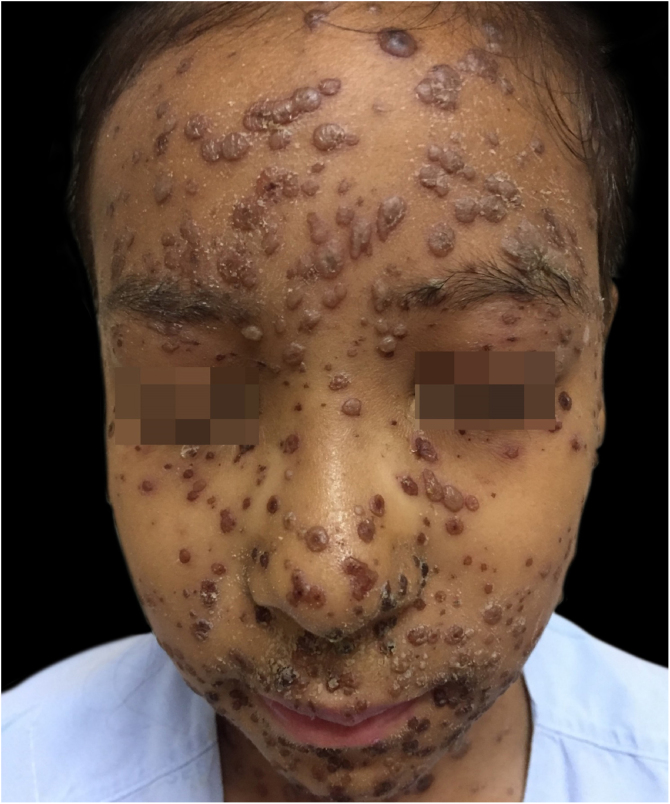
At the seventh day of intravenous acyclovir, with a regressing bullous eruption and crusts.

**Figure 4:**
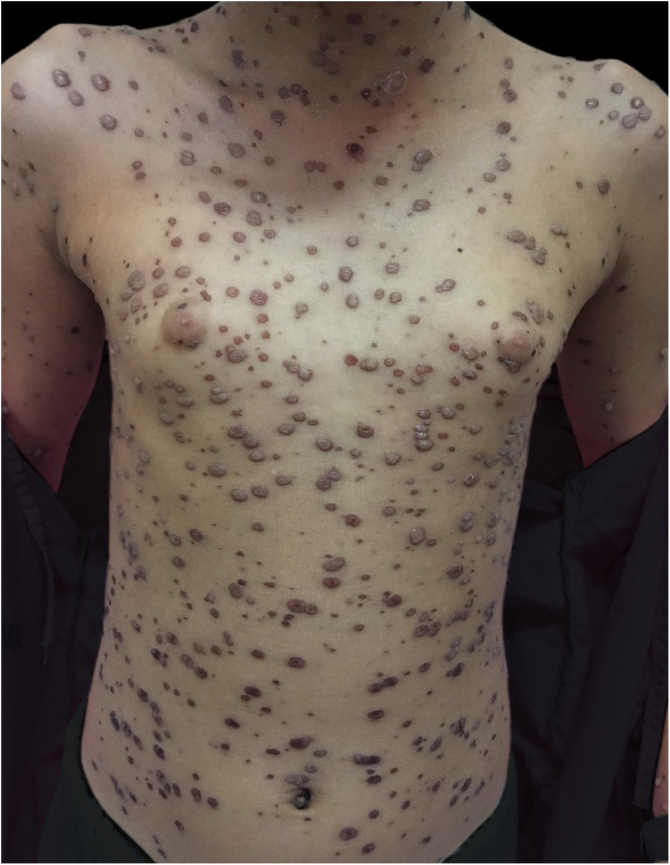
At the seventh day of hospital admission, with a regressing bullous eruption and crusts.

During hospitalization, the disease evolved with the dissemination of lesions that exceeded a 1,000 count, periorbital cellulitis, and febrile neutropenia, so cefepime was prescribed (Fig. 2). Folinic acid was also started to improve the effects of methotrexate, as well as dexchlorpheniramine for pruritus. After six days of hospitalization, the fever ceased, and the lesions started to regress, and the combination of hypochlorous acid and silicone was started for better healing (Figure 3, Figure 4).

After ten days of intravenous acyclovir use, the patient was discharged, with most of the skin lesions showing crusts. She was instructed to maintain methotrexate withdrawal, use cephalexin for another five days, as well as maintain the use of hypochlorous acid with silicone and baths with a potassium permanganate solution.

**Figure 5:**
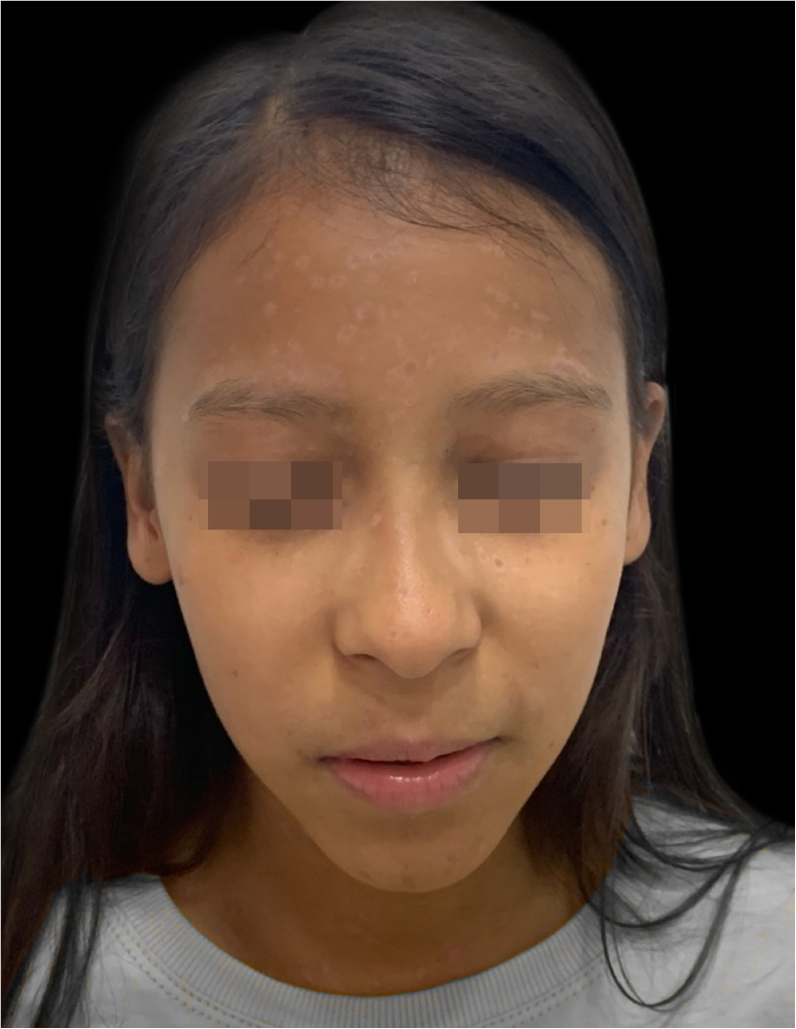
Sixty days after hospital discharge, with residual hypochromic spots.

Despite the severity of the initial condition, she showed a good evolution, with excellent healing of the lesions (Fig. 5).

## Discussion

The child, who had erythrodermic psoriasis and had been using methotrexate for one year and five months without being previously vaccinated against varicella, developed a severe form of the disease. During evolution, two important complications occurred: periorbital cellulitis and febrile neutropenia. The case illustrates how infections in immunosuppressed individuals can rapidly progress to severe complications.

There are well-established recommendations regarding vaccination in children with rheumatologic diseases undergoing treatment with immunosuppressants.^8^ Overall, it is recommended to maintain the national vaccination schedule updated. Data on children treated with methotrexate who received live attenuated virus vaccines are reassuring regarding the safety and immune response to the vaccine.^6^, ^9^ There are studies that specifically mention methotrexate and the varicella vaccine: in cases of screening to start treatment with methotrexate in a child who is not immune to varicella, it is recommended to administer the vaccine and wait two to four weeks before starting the medication.^8^ However, the data in the literature are scarce regarding dermatological diseases.

In Brazil, there are no clear recommendations on how to proceed regarding vaccination with a live attenuated virus in children with psoriasis using methotrexate,^10^ and it is not part of the routine to update the vaccination card before starting the medication. In the Brazilian context, this issue becomes even more relevant due to the history of the varicella vaccine: it was included in the national vaccination schedule of the SUS only in 2012, for children aged 15 months; therefore, there is a large contingent of the population without specific immunity against the varicella-zoster virus.

Considering the reported case and its severity, it is necessary to alert about the need to consider the vaccination status before starting treatment with methotrexate. For non-immune children who are already undergoing treatment, assessing the degree of immunocompromise and epidemiological risk can guide the decision-making.

## Financial support

None declared.

## Authors' contributions

Letícia Oba Galvão: Effective participation in research orientation; intellectual participation in the propaedeutic and/or therapeutic conduct of the studied case; drafting and editing of the manuscript; critical review of the manuscript; approval of the final version of the manuscript.

Carmelia Matos Santiago Reis: Effective participation in research orientation; intellectual participation in the propaedeutic and/or therapeutic conduct of the studied case; design and planning of the study; critical review of the manuscript.

Natália Lima Alves: Collection, analysis, and interpretation of data; design and planning of the study; drafting and editing of the manuscript; critical review of the literature; critical review of the manuscript.

Elisa Scandiuzzi Maciel: Collection, analysis, and interpretation of data; design and planning of the study; drafting and editing of the manuscript; critical review of the literature; critical review of the manuscript.

## Conflicts of interest

None declared.
